# Greenlight laser (XPS-180watt) prostatectomy for treatment of benign prostate obstruction, Pursuit of durability

**DOI:** 10.1080/2090598X.2023.2220631

**Published:** 2023-06-09

**Authors:** Fady K. Ghobrial, Mahmoud Laymon, Nasr El-Tabey, Ahmed M. Elshal

**Affiliations:** a Department of Urology, Urology and Nephrology Center, Mansoura University, Mansoura, Egypt; b Department of Urology, Faculty of Medicine, Damietta University, New Damietta, Egypt

**Keywords:** Transurethral prostatectomy, greenlight, BPH, prostate, vaporization

## Abstract

**Objectives:**

To report 5-year outcomes, need and predictors of retreatment post greenlight laser photoselective vaporization (GL.PVP) and vapo-enucleation (GL.PVEP), as long-term data on safety and efficacy of GL.PVP and GL.PVEP and on the prostate using XPS^TM^ system are still pending.

**Patients and methods:**

Primary outcome was the need for retreatment (medical treatment and reintervention) for recurrent BOO. Time-to-event (retreatment) analysis, perioperative events, change in the urinary outcome measures at different follow-up visits, early and late complications and PSA kinetics were reported.

**Results:**

Between September 2014 and April 2017, 248 patients underwent GL/XPS procedures. GL.PVP and GL.PVEP were carried out for 157 (63.3%) and 91 (36.7%) patients with mean prostate sizes of 60 ± 18 and 100 ± 22 cc, respectively. After a mean duration of 62 ± 9-month follow-up, overall retreatment rate (medical and interventional) was 23% (57 patients). It was comparable between both GL.PVP and GL.PVEP cases: 38 (24.2%) and 19 (20.9%) patients, *P* = 0.5, respectively. Significantly more surgical reintervention rate was reported after GL.PVP compared to GL.PVEP (*P* = 0.03). In retreatment group, more intraoperative bleeding (*P* = 0.02), early postoperative hematuria (*P* = 0.03), higher median preoperative PSA (*P* = 0.02) and less postoperative one-year percent PSA reduction (*P* = 0.02) were detected. Lower postoperative one-year percent PSA reduction independently predicts retreatment with a cut-off point of 64.2% (58.2% sensitivity, 73.4% specificity, AUC 0.647, 95% CI 0.52–0.76).

Median (range in months) time to event was 20 (1–60) for all cases and 13.5 (1–42) and 30 (18–60), *P* = 0.7, for GL.PVP and GL.PVEP groups, respectively.

**Conclusion:**

Greenlight laser XPS is an effective, durable and versatile tool in treating benign prostatic obstruction. Durability of the outcome is predictable with more postoperative PSA reduction.

## Introduction

Lower urinary tract symptoms (LUTs) due to benign prostatic obstruction (BPO) negatively impact patients’ quality of life. TURP is considered the cornerstone of BPO treatment particularly for small- and medium-sized prostate, while holmium laser enucleation of the prostate (HoLEP) or open prostatectomy (OP) are the gold standard for treatment of large-sized BPO [1]. However, TURP and OP are associated with safety concerns regarding intraoperative and postoperative complications and particularly for patients with coagulopathy [2,3]. Hence, rise of the use of laser-based minimally invasive procedures has a dramatic effect on patient’s safety [4]. Over the past decade, three generations of greenlight laser (532 nm) have been commercialized and finally greenlight laser XPS [5]. Greenlight laser XPS (GL/XPS) 180-Watt is characterized by a high degree of safety and reliability by higher energy limits and enhanced Moxy fiber criteria, obtaining better vaporization efficiency even in high-risk patients [6,7]. The versatility of greenlight laser makes it more flexible for different surgical techniques and surgeons’ skills [8]. Long-term data on safety and efficacy of greenlight laser XPS are still pending [9,10]. Two-year outcome of Goliath trial was reported, showing that greenlight laser XPS photoselective vaporization of the prostate (GL.PVP) has similar safety and efficacy compared to TURP for control of LUTs secondary to BPO with size ≤100 ml [11]. Three-year outcome of Greenlight XPS photoselective vapo-enucleation of the prostate (GL.PVEP) was reported for treatment of large BPO with size more than 80 ml using precepts of enucleation followed by vaporization of partially enucleated adenoma without a need for morcellation [12].

As greenlight laser is an evolutional laser modality, long-term durability and efficiency measures should be tested to better define its role among BPO treatment lines. Calves et al. reported a mean 57-month follow-up for 84 patients after GL.PVP focusing only on patient-reported questionnaire, lacking objective urinary outcome measures [13]. Ajib et al. reported a 5-year follow-up post GL.PVP for 66 patients with prostate size below 80 ml [14].

Herein, we will report a 5-year outcome, need for retreatment as well as predictors of retreatment post of GL.PVP and GL.PVEP.

## Patients and methods

### Study design

After IRB approval (R.23.02.2056), our prospectively maintained database was reviewed for patients who had GL/XPS procedures (AMS/Boston Scientific, Marlborough, MA, USA) with side-firing fiber (MoXy^TM^) before April 2017. Patients were invited for updated follow-up visits and were asked to sign an informed written consent.

### Study population

Patients included in the study had GL/XPS procedures either as a part of a randomized trial [15,16] or according to patient or surgeon preference. The procedures were done by one of two experienced surgeons (AME and ML). Legible patients for BPO treating procedures were included in which an upper limit for estimated prostate size of 150 ml was set as an institutional guideline for GL/XPS procedures.

Patients with neurological disorders (neurological deficit, poor anal tone and or non-reactive bulbocavernosus reflex) or diagnosed with prostate cancer were excluded from the study.

### Study work-up

Patients were assessed preoperatively by International Prostate Symptoms Score (IPSS), quality of life (QoL), uroflow (Q max), post-void residual (PVR), transrectal ultrasound (TRUS) and prostate specific antigen (PSA) at the time of enrollment. Prescheduled clinic visits at 3, 6 and 12 months postoperatively and then annually were set for assessment of IPSS-QoL, uroflow and PVR. PSA measurement was done at 1 year postoperative and then annually. Patients reporting recurrence of obstructive LUTs were offered alpha-blockers and response was reassessed in 2–4 weeks afterwards. Outpatient diagnostic cystoscopy was carried out if patient experienced resilient bothersome LUTs and/or obstructed flow rate during follow-up. If there was a definite obstruction, re-intervention for recurrent BOO was planned accordingly.

### Surgical technique

In both procedures, a 26-Ch continuous flow resectoscope was used. The procedure starts by creation of a working channel and vaporization of median lobe, if present. For GL.PVP, a standard technique [17] was carried out. In cases of GL.PVEP, the adenoma is bluntly enucleated as previously described [12] after creation of the working channel. Both lateral lobes were partially enucleated by sweeping underneath the adenoma. Ablation of the adenoma was conducted with 180 W power. Finally, clearing apical tissue was carefully performed to avoid damage to the sphincter ending up with TURP-like cavity lined with capsular fibers and tongue-like projection of the verumontanum for both procedures.

### Outcomes measures

The primary outcome was the need for retreatment for recurrent BOO (study’s endpoint). Retreatment entails reuse of prostate-targeted medicines for regrowing or residual BPO or reintervention for regrowing or residual BPO, bladder neck contracture (BNC) or urethral stricture.

Secondary outcomes include time-to-event analysis. Event was defined as time to retreatment for recurrent BOO either medical or reintervention. Furthermore, perioperative events, change in the urinary outcome measures (IPSS-QoL, uroflow and PVR) at different follow-up visits, early and late complications and PSA kinetics (percent change from baseline) were reported.

### Statistical analysis

Data analysis was conducted using a commercially available SPSS®20. Descriptive statistics were reported in terms of number (percentages) or medians (range)/means (SD) for categorical and continuous variables, respectively. A chi-square test was used to compare categorical variables, and Student’s t-test or Mann–Whitney U-test was used as appropriate to compare continuous variables. Univariate, multivariate and regression analyses were done as indicated. A critical two-sided *P* value < 0.05 was used for statistically significant differences. Receiver operating characteristic (ROC) curve was used whenever indicated.

## Results

Between September 2014 and April 2017, 248 patients underwent GL/XPS procedures. GL.PVP and GL. PVEP were carried out for 157 (63.3%) and 91 (36.7%) patients with mean preoperative estimated prostate sizes of 60 ± 18 and 100 ± 22 cc, respectively. After a mean duration of 62 ± 9-month follow-up, overall retreatment rate (medical and interventional) for recurrent BOO was 23% (57 patients). Table 1 summarizes baseline and perioperative parameters for the whole cohort, non-retreatment (group I) and recurrent BOO requiring retreatment group (group II).

**Table 1.** Baseline and perioperative parameters.

	Overall (248)	Group I (191)	Group II (57)	*P* value
Mean ± SD age (years)	64 ± 7.4	64 ± 7	64 ± 7	0.5
Median (range) ASA score	2 (1–3)	2 (1:3)	2 (1:3)	0.5
No. of patients with indwelling urethral catheter (%)	87 (35)	72 (37.7)	15 (26.3)	0.1
Median (range) of duration of indwelling catheter (months)	3 (0.5–32)	3 (0.5:32)	5 (1:24)	0.2
No. of patients with diabetes mellitus (%)	66 (26.6)	52 (27.2)	14 (24.6)	0.7
No. of patients with bleeding tendency (%)	43 (17.3)	34 (17.8)	9 (15.8)	0.7
No. of patients on antiplatelet (%)	11 (4.4)	10 (5.2)	1 (1.8)	0.2
Mean ± SD IPSS	25 ± 5.6	25 ± 6	25.5 ± 4	0.8
Mean ± SD QoL	5 ± 1	4 ± 1	5 ± 0.9	0.4
Mean ± SD Q max	9 ± 3.5	9.4 ± 3.4	8.3 ± 3.7	0.2
Median (range) PVR	21 (0–306)	22 (0:306)	20 (0:120)	0.4
Median (range) PSA (ng/ml)	4 (0.3–73)	3.6 (0.3:73)	5.2 (0.9:16.5)	0.02
Mean ± SD TRUS (ml)	75 ± 27	75 ± 27	75 ± 26	0.9
Mean ± SD Total energy (kJ)	379 ± 162	381 ± 155	374 ± 186	0.8
Mean ± SD Operative time (min)	77 ± 35	78.2 ± 35	74 ± 36	0.4
Mean ± SD Lasing time (min)	44 ± 20	45 ± 20	42 ± 19	0.2
Mean ± SD Vaporizing efficiency (kJ/ml)	5.5 ± 2.5	5.5 ± 2.4	5 ± 2.3	0.4
Median (range) Catheterization time (days)	1 (1–5)	1 (1:5)	1 (1:5)	0.2
Median (range) Hospital stay (days)	1 (1–10)	1 (1:10)	1 (1:10)	0.3
No. of patients with intraoperative bleeding (%)	2 (0.8)	0	2 (3.5)	0.02
No. of patients with postoperative hematuria (%)	8 (3.2)	3 (1.6)	5 (8.7)	0.03
No. of patients with failed 1st TOV (%)	7 (2.8)	6 (3.1)	1 (1.8)	0.7
No. of patients with AUR during 1st month (%)	4 (1.6)	2 (1)	2 (3.5)	0.1
Median % (range) postoperative PSA reduction at 1 year	63.6 (−75:99)	67.1 (−72:99)	54.3 (−75:96)	0.02

Group I, no retreatment; Group II, recurrent BOO with retreatment; ASA, American Society of Anesthesiologists; TRUS, transrectal ultrasound; IPSS, International Prostate Symptoms Score; QoL, quality of life; Q max, maximal flow rate; PVR, post-void residual urine, vaporizing efficiency; utilized laser energy in kJ divided by estimate prostate size in ml.

Early and late complications are summarized in Table 2 according to Modified Clavien–Dindo classification.

**Table 2.** Perioperative and delayed postoperative complications.

	Clavien–Dindo grade	Management	Overall (248)
Perioperative (intraoperative and first 30 days) No. (%)			45 (18.1)
Fever (UTI)	I	Fomentations & antipyretics	4 (1.6)
Capsular violation	II	Prolonged catheter drainage	10 (4)
Bladder wall injury	II	Prolonged catheter drainage	4 (1.6)
Failed 1st TOV	II	Catheterization & repeat voiding trial	7 (2.8)
AUR during 1st month	II	Catheterization & repeat voiding foments	4 (1.6)
Epididymo-orchitis	II	Antibiotics & lead-subacetate foments	3 (1.2)
Intraoperative bleeding	IIIa	Coagulation using TUR loop	2 (0.8)
Urosepsis	IVa	Systemic antibiotics & ICU measures	3 (1.2)
Post-operative hematuria	II	Prolonged CBI	7 (2.8)
Post-operative hematuria	IIIa	Cystoscopy and hemostasis	1 (0.4)
Delayed postoperative (after 30 days) No. (%)			60 (24.2)
Recurrent BOO No. (%)			57 (23)
○ Residual obstructive LUTs	II	Resume alpha blockers; control LUTs	40 (16.1)
○ LUTs with recurring prostate adenoma	IIIa	Redo for adenoma (TURP)	13 (5.2)
○ Bladder neck contracture	IIIa	Bladder neck incision	2 (0.8)
○ Urethral stricture	IIIa	Internal urethrotomy	2 (0.8)
Persistent stress urine incontinence	IIIa	Midurethral sling	3 (1.2)

UTI, urinary tract infection; TOV, trial of voiding; AUR, acute urine retention; BOO, bladder outlet obstruction; LUTs, lower urinary tract symptoms; CBI, continuous bladder irrigation.

Overall retreatment was comparable between both GL.PVP and GL.PVEP cases: 38 (24.2%) and 19 (20.9%) patients, respectively (*P* = 0.5). Medical retreatment in the form of alpha-blocker was required for 23 (14.6%) and 17 (18.7%) patients, *P* = 0.4, post GL.PVP and GL.PVEP, respectively. However, a significantly more reintervention (surgical) rate for recurrent BOO was reported after GL.PVP compared to GL.PVEP: 15 (9.6%) vs. 2 (2.2%) patients (*P* = 0.03) (Table 3).

**Table 3.** Details of retreatment.

	Overall (248)	GL.PVP (157)	GL.PVEP (91)	*P* value
Retreatment for recurrent BOO. No. (%)	57 (23)	38 (24.2)	19 (20.9)	0.5
Medical treatment (α-blockers). No. (%)	40 (16.1)	23 (14.6)	17 (18.7)	0.4
Re-intervention. No. (%)	17 (6.9)	15 (9.6)	2 (2.2)	**0.03**
No. TURP for residual adenoma (%)	13 (5.2)	11 (7)	2 (2.2)	0.1
No. BNI for BNC (%)	2 (0.8)	2 (1.3)	0	0.2
No. DVIU for urethral stricture (%)	2 (0.8)	2 (1.3)	0	0.3

BOO, bladder outlet obstruction; TURP, transurethral resection of the prostate; BNI, bladder neck incision; BNC, bladder neck contracture; DVIU, dorsal visual internal urethrotomy.

In retreatment group, more intraoperative reported bleeding (0.02), early postoperative hematuria (*P* = 0.03), higher median preoperative PSA (*P* = 0.02) and less postoperative one-year percent PSA reduction *P* = 0.02 were reported. On multivariate analysis, lower postoperative one-year percent PSA reduction was a significant independent predictor for overall retreatment (surgical/medical) for recurrent BOO (*P* 0.021, 95% CI 0.018–0.024).

**Figure 1.**
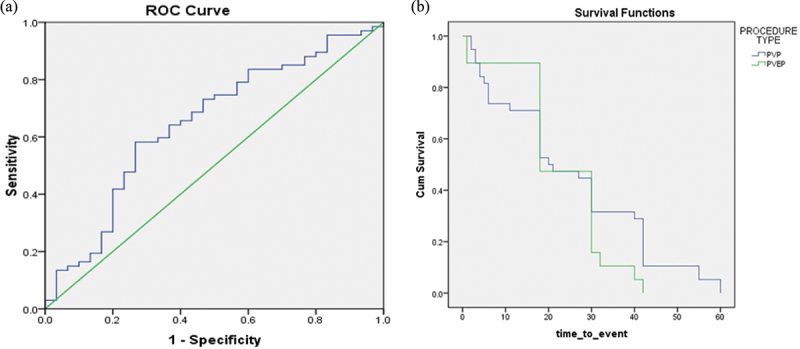
a) ROC curve for postoperative percent PSA reduction. b) Time to event (retreatment).

The cut-off point for percent reduction of one-year post-operative PSA associated with less probability for having retreatment for recurrent BOO was 64.2% (58.2% sensitivity, 73.4% specificity, AUC 0.647, 95% CI 0.52–0.76) (Figure 1a).

Median (range in months) time to event (retreatment for recurrent BOO) was 20 (1–60) for all cases and 13.5 (1–42) and 30 (18–60), *P* = 0.7, for GL.PVP and GL.PVEP groups, respectively (Figure 1b).

Table 4 summarizes types of retreatments and their timing.

**Table 4.** Time to retreatment.

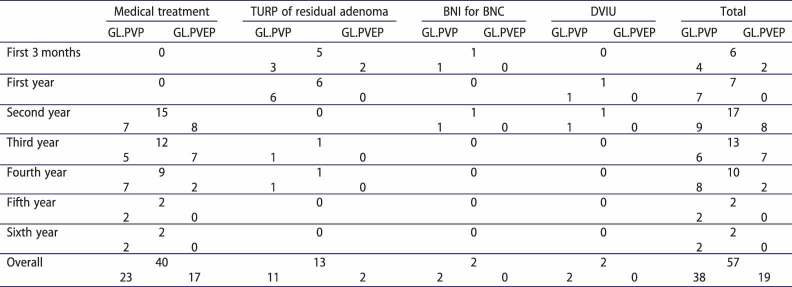

TURP, transurethral resection of the prostate; BNI, bladder neck incision; BNC, bladder neck contracture; DVIU, dorsal visual internal urethrotomy.

**Figure 2.**
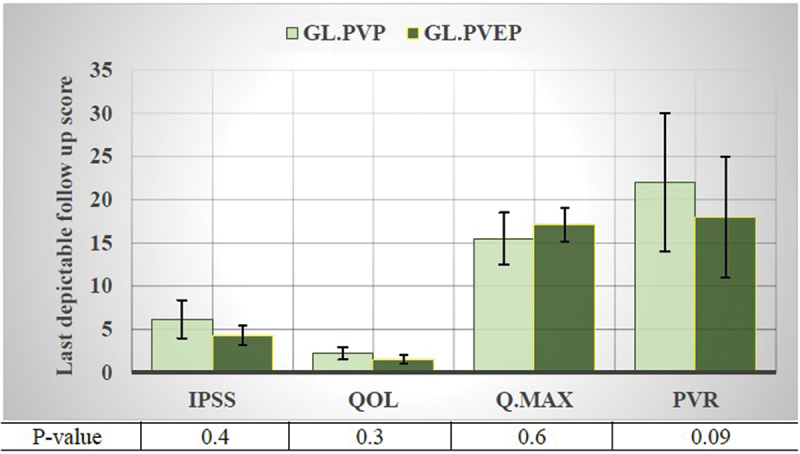
Urinary functional outcome measures at last depictable follow-up. Error bars, standard errors of the mean.

At last depictable follow-up, mean ± SD percentage of improvement in IPSS was 61 ± 28 and 78 ± 20% *P* = 0.03, median (range) percentage of improvement in Q max was 119 (−13–549) and 123 (−13–649)% *P* = 0.2 and median (range) percentage of PSA reduction was 63 (−72–99) and 65 (−75–97)% *P* = 0.7 for GL.PVP and GL.PVEP groups, respectively. Figure 2 summarizes the last depictable follow-up IPSS and QoL, Q max and PVR in GL.PVP and GL.PVEP.

## Discussion

Durability of clinical efficacy and prospering the test of time are the main requirements for any new gold standard surgical tool. Favorable outcomes of GL-XPS for the treatment of BPO are evident, with comparable functional results, less morbidity, and shorter catheterization and hospitalization time compared with TURP; however, long-term studies are sparse [10]. The non-inferiority of GL-PVP in Goliath trial was limited to moderate prostate sizes (median 48 cc), and efficacy of GL-PVP in larger adenomas is questionable. Furthermore, the favorable clinical outcomes reported at 2 years need to be assessed and reported at 5 years [11]. Long-term follow-up duration for clinical studies – as defined by EAU – should be more than 36 months. In the current study, a mean ± SD duration of 62 ± 9-month follow-up for 248 GL-XPS procedures was reported with median ± SD prostate volume of 75 ± 27 cc.

A systematic review of 1640 patients who underwent GL.PVP XPS reported median IPSS score between 5 and 9.5 in a short-term follow-up [10]. The current study showed a median (range) IPSS score of 4 (1–10) at last follow-up compared to 5.9 ± 5 as reported by Calves et al., a long-term report [13]. An improvement in IPSS score at 12 months by 79.6% was reported after GL.PVEP [12]; likewise, in the current study, 78% IPSS improvement was reported at last follow-up, indicating long-term BPO-related LUTs control.

Modified Clavien–Dindo score has been utilized for categorization of post GL.PVP complications. However, most of the complications are of minor degree [18]. Similarly, in the current study, postoperative events were 105 reported in 78 patients (31.4%); 45 (18.1%) patients had grade I–II and 33 (13.3%) patients had grade III–IVa complications. Similarly in Goliath trial, 22 (16%) patients had grade III complications [11].

Calvas et al. reported an overall retreatment rate of 17.8% (15/84 patients) following GL.PVP after a mean follow-up of 57.4 ± 6 months [13]. In their study, surgical retreatment for bladder neck contracture, urethral stricture and recurring prostate adenoma was 2.4% for each, alpha-blockers reuse was 5.9% and retreatment for post GL.PVP incontinence was 4.7%.

Campobasso et al. reported on 867 GL.PVP procedures done in 20 Italian centers with a median follow-up period of 32.5 months (interquartile range: 20–49 months), with 6.1% (53/867) reintervention rate; 18 urethral stricture, 22 bladder neck or prostatic fossa contracture and 13 patients had redo for recurring symptomatic adenoma. Larger prostate volume ≥100 ml, preoperative urethral stricture and occurrence of early complications correlated with re-intervention [19]. The diversity of centers and surgeons with long recruitment period (2011–2019) weakens their conclusions furthermore, and no data on medical retreatment were reported.

Ajib et al. reported on 370 single-surgeon GL.PVP procedures with 10 (2.7%) conversion rate. In the first 12 months, bladder neck contracture and urethral stricture were reported in seven and three cases, respectively. Reintervention for recurring adenoma was reported in 2/144 and 2/42 of evaluable subjects at 24 and 48 months, respectively [20]. No data were reported on the need for medical retreatment. Retreatment rate in the current study was 22.9% (57/248 patients) after a mean duration of 62 ± 9-month follow-up. Reporting medical retreatment (16.1%) and long-term follow-up could explain the higher retreatment rate.

Cornu et al., in their meta-analysis, reported 70% improvement in IPSS at a 5-year follow-up after TURP [21], which is similar to 61% ± 28% and 78 %± 20% improvement reported in the current study after GL.PVP and GL.PVEP, respectively. Following bipolar TURP, reoperation for regrowing adenoma and urethral stricture was reported in 9% and 4% of the cases, respectively, at 100-month follow-up [22]. Xie et al. reported 5-year follow-up post TURP in which 2.7%, 3.6%, and 7.3% of the patients were reoperated for adenoma regrowth, urethral stricture and bladder neck contracture, respectively [23]. Efficacy of GL.PVEP was not inferior to HoLEP considering urinary functional outcomes with 3.7% and 2% reoperation rate for recurrent BOO, respectively, at 1-year follow-up [12].

In the current study, re-intervention for BOO was significantly higher post GL.PVP group than GL.PVEP and almost all cases needed redo-TURP in the first year postoperatively while medical retreatment was indicated mainly starting from the second year postoperatively for both groups.

Valdivieso et al. suggested a long-term follow-up for PSA reduction for evaluation of the efficacy and durability of GL.PVP XPS [24]. The current study proved that long-term PSA reduction could be considered as a surrogate marker for a need for retreatment. This correlates with previous studies on GL.PVP systems [25] and adenoma recurrence 10 years after HoLEP [26]. PSA reduction is affected by amount of energy delivered to prostate tissue, reflecting more adenoma removal [24]. In this study, median postoperative percent PSA reduction for the whole cohort was 64%, similar to what was reported by Misrai et al. [27], with a PSA reduction from 37% to 67% during completion of the learning curve of GL.PVP XPS.

Meshkawi et al. [28] analyzed the outcome of 438 patients treated with GL.PVP XPS, with median prostate volume of 135 ml. They reported surgical retreatment for regrowing adenoma in 15.6% compared to 2.2% (2 of 91 cases) in the current study for GL.PVEP group. They explained this higher retreatment rate by larger adenoma and less energy density of 2.4 kJ/cc in the retreatment group added to lower postoperative percent PSA reduction at 1 year (median 17.5%). They recommended utilizing a standardized surgical technique (enucleation-like-defect) and an optimized energy density >3 kJ/cc to overcome the adverse effects of vaporization technique alone for large-sized prostate.

The main limitations of the current study are the lack of sexual function assessment and heterogeneity of inclusion criteria for study’s subjects. Nevertheless, to the best of our knowledge, this is the first long-term study done in a single center with prospectively maintained database reporting all kinds of retreatments.

## Conclusion

GL/XPS is an effective, durable and versatile tool in treating BPO. The durable efficacy of GL.PVP and GL.PVEP techniques suggests that tailoring the technique according to the prostate size is saving health and wealth. Durability of the outcome is predictable with more postoperative PSA reduction.

## Abbreviation list

AUR: acute urine retention
BNC: bladder neck contracture
BOO: bladder outlet obstruction
BPO: benign prostatic obstruction
GL.PVEP: Greenlight XPS photoselective vapo-enucleation of the prostate
GL.PVP: greenlight laser XPS photoselective vaporization of the prostate
GL/XPS: greenlight laser extreme power system
HoLEP: holmium laser enucleation of the prostate
IPSS: International Prostate Symptoms Score
IRB: institutional review board
LUTs: lower urinary tract symptoms
OP: open prostatectomy
PSA: prostate specific antigen
PVR: post-void residual
Q.max: maximum uroflow
QoL: quality of life
TOV: trial of voiding
TRUS: transrectal ultrasound
TURP: transurethral resection of the prostate
